# Resorption of roots in conventional braces versus advanced aligners: A comparative study

**DOI:** 10.6026/973206300221318

**Published:** 2026-03-31

**Authors:** Saloni Pansotra, Ravi Ranjan, Jain Mathew, Robin Theruvil, Bijo Kurian, Renu Ann Mathew

**Affiliations:** 1 Department of Orthodontics and Dentofacial Orthopedics, Rayat Bahra Dental College and Hospital, Rayat Bahra University, Mohali, India; 2 Department of orthodontics, Sido Kanhu Medical College and Hospital, Dumka, Jharkhand, India; 3 Department of Conservative Dentistry and Endodontics, St Gregorios Dental College, Chelad, Kerala, India; 4 Department of Conservative Dentistry and Endodontics, Al Azhar Dental College, Thodupuzha, Idukki, Kerala, India; 5 Department of Pedodontics, Al Azhar Dental College, Thodupuzha, Idukki, Kerala, India

**Keywords:** Orthodontic, root resorption, clear aligner, fixed appliance, radiographic evaluation

## Abstract

External apical root resorption (EARR) remains a common and clinically relevant iatrogenic consequence of orthodontic treatment and uncertainty persists regarding whether modern clear aligner systems reduce this risk compared with conventional fixed appliances. Therefore, it is of interest to evaluate the radiographic alterations in the root length of patients using conventional braces to patients with the new generation aligners. There was a significant resorption in both groups although it was a mild one in clinicallee where the mean reduction is more in patients with fixed appliances. Data show evidence in favor of aligner therapy as a possible option in reducing orthodontic roots resorption.

## Background:

External apical root resorption can be an iatrogenic, multifactorial condition that is caused by orthodontic force use and the individual susceptibility to biological factors. Continuous or excessive forces applied in fixed appliance therapy are associated with a more resorption in vulnerable patients [1, 2]. Recent advancements in clear aligner technology allow controlled intermittent forces and stages of tooth movement; possibly lower the risk of resorption [3]. Cone-beam computed tomography as well as digital radiography of the periapical area has enhanced the detection of tiny changes in the length of the root in the course of treatment [4]. Clinical evidence is still insufficient about the different resorption outcomes of modern aligner protocols as well as conventional bracket systems [5]. Therefore, it is of interest to report and compare the extent and pattern of external apical root resorption in patients treated with conventional fixed appliances versus advanced clear aligner therapy using standardized radiographic measurements.

## Materials and Methods:

Prospective comparative clinical study conducted in an orthodontic department. Forty patients in need of non-extraction comprehensive orthodontic treatment were selected according to ethical considerations and obtained written consent. Conventional 0.022-inch slot MBT prescription fixed appliances were used to treat 20 patients and advanced clear aligner therapy with staged movement protocol was intended for other twenty patients. The inclusions were the permanent dentition, mild to moderate crowding and absence of root pathology. Exclusion criteria consisted of a history of orthodontic therapy, systemic disease with implications for bone remodeling and dental trauma. Periapical radiographs from maxillary and mandibular incisors were taken at pre-treatment (T1) and post treatment. The length of the roots was measured and analysed by image analysis software. The reduction in mean root length was measured in millimetres. Differences between groups were compared using independent t-tests and significance was set at p<0.05.

## Results:

Maxillary incisors showed an external apical root resorption that was measurable within both groups of treatment. The fixed appliance group demonstrated a larger reduction in length of the root as compared to an aligner-only group. A statistical analysis showed that there was a significant distinction between the two groups (p=0.003). Although resorption was evident radiographically, the magnitude remained within clinically acceptable limits. None of the patients showed a severe resorption more than a third of the initial root length. These results show that there are less resorptive changes in relation to aligner-mediated force systems. Table 1 (see PDF) The pattern of resorption of mandibular incisors resembled that of maxillary teeth. The mean shortening in the root of patients undergoing fixed appliances was also significantly higher than those of patients undergoing clear aligners (p=0.01). This difference may be due to the lesser scale of force loading and the alternating activity of aligner therapy. There was no statistically significant relationship between the duration of the treatment and the severity of the resorption. In general, the value of resorption of mandibular incisors was lower than maxillary incisors in both groups Table 2 (see PDF).

## Discussion:

Both traditional fixed braces and modern clear aligners in this research led to the external apical root resorption (EARR) that could be measured. Nevertheless, shortening of roots was evidently greater in the fixed appliance group. The significance of this finding is that EARR is among the most prevalent adverse effects of orthodontics. It may have an impact on long-term tooth stability particularly the maxillary incisors. The nature of force delivery is one of the factors that made fixed appliances to have a greater resorption. The forces caused by the braces are usually continuous and the movement of the teeth can still proceed even when the patient is not regularly visiting. Aligners tend to offer more precise and graduated movement. They also enable intermittent forces since the aligners will be taken off during meals and cleaning. Intermittent force systems tend to be safer in as far as root structure and periodontal tissues are concerned. The current clinical evidence justifies this explanation and states that EARR severity can be lowered by aligners in comparison to fixed appliances [6]. The current findings are also in line with more recent studies that EARR remains possible with aligners although it is often mild and can be considered clinically acceptable. A number of studies in the recent years have also reported that aligner therapy may still lead to resorption, particularly in the incisors, since they tend to be tipped, torqued and intruded during the alignment process. The extent of resorption is however, in most instances less than that of the case of braces [7]. This is in line with our results because none of the patients had severed resorption exceeding a third of the initial root length. Maxillary incisors exhibited greater shortening of the roots as compared to mandibular incisors in the two groups of our study. This trend is widely documented in dental orthodontic literature. The roots of the maxillary incisors are thinner and they are more susceptible to stress during retraction, torque and leveling. In recent studies conducted on radiographic and clinical evaluation, it has been indicated that the upper incisors are still the most susceptible to EARR, despite the current aligner guidelines [8]. This justifies the fact that our maxillary outcomes were stronger. A note that should be mentioned is that force alone does not lead to root resorption. Major roles are played by biological susceptibility. Patients vary in their inflammatory reaction, morphology of roots and genetic risk. This explains why mild resorption is exhibited by some patients despite the low forces. Even with controlled treatment, other patients can exhibit the increased resorption. Current findings have revealed the role of personal risk factors and the necessity of close observation, irrespective of the type of appliances [9]. The other contribution of the literature is the role of imaging. Periapical radiographs are commonly applied and they might not detect subtle resorption. CBCT is able to pick smaller changes and the patient has increased exposure to radiation. The current research indicates that radiographs can be used in regular monitoring, whereas CBCT is recommended to be applied to select cases with high risk or ambiguous results [10]. We applied standard radiographs in our approach, which is clinically convenient. The clinical implication of the present study is that aligners can potentially provide a safer alternative to patients with the potential of developing EARR like patients with thin roots or prior trauma. More recent comparative reports demonstrate inferior average resorption in cases of aligner treatment [11, 12-13]. Nevertheless, professionals should keep in mind that aligners are not entirely safe. The level of movement intended, particularly intrusion and torque ought to be well regulated [14]. These two types of appliances should be monitored, particularly among incisors and long procedures [15]. Comprehensively, our results confirm the current evidence of the superiority of advanced aligners in the decrease of the average severity of EARR, relative to traditional braces, but with the need to conduct appropriate case selection and follow-up.

## Conclusion:

The conventional braces as well as the advanced aligners have demonstrated significant external apical root reduction. Fixed appliance therapy showed significantly more root shortening when compared to aligner treatment. Advanced aligner procedures are able to decrease the risk of resorption while maintaining efficient dental tooth movement.

## Advancement to knowledge:

This study adds recent (2020-2026) comparative clinical evidence demonstrating that advanced clear aligner therapy is associated with significantly lower mean external apical root resorption than conventional fixed appliances under standardized radiographic assessment, thereby strengthening contemporary evidence that force modulation and intermittent biomechanics influence EARR severity.

## References

[1] Li Y (2020). Prog Orthod..

[2] Toyokawa-Sperandio KC (2021). Korean J Orthod..

[3] Liu W (2021). Angle Orthod..

[4] Gandhi V (2021). Eur J Orthod..

[5] Yassir YA (2022). Clin Oral Investig..

[6] Almagrami I (2023). Prog Orthod..

[7] Singh S (2024). J Dent Res Dent Clin Dent Prospects..

[8] Butsabul P (2024). Int Dent J..

[9] Selvaraj M (2025). J Oral Biol Craniofac Res..

[10] Devizes A (2025). Dent J (Basel)..

[11] Kreuter P (2025). BMC Oral Health..

[12] Kurnaz S, Buyukcavus MH (2024). BMC Oral Health..

[13] Rossi A (2024). Angle Orthod..

[14] Al-Rokhami RK (2025). Sci Rep..

[15] Bayir F, Gumus ES (2021). J Dent Res Dent Clin Dent Prospects..

